# Incidence of uveitis and macular edema among patients taking fingolimod 0.5 mg for multiple sclerosis

**DOI:** 10.1186/s12348-020-00215-1

**Published:** 2020-09-21

**Authors:** Scott Joseph Sonne, Bradley Thomas Smith

**Affiliations:** 1 St. Louis University School of Medicine, St. Louis, USA; 2 The Retina Institute in St. Louis, 2201 S Brentwood Blvd, St. Louis, MO 63144 USA; 3 Department of Ophthalmology and Visual Sciences, Washington University, St. Louis, USA

**Keywords:** Cystoid macular edema, Macular edema, Uveitis, Multiple sclerosis, Acute macular neuroretinopathy, Fingolimod, Gilenya

## Abstract

**Background:**

Patients with multiple sclerosis (MS) have a higher incidence of uveitis compared with the general population. Fingolimod, a first line disease modifying drug used in multiple sclerosis, may cause macular edema and thus requires ophthalmic examination. However, murine models and anecdotal reports suggest fingolimod may reduce the incidence of uveitis.

**Purpose:**

To report the incidence of uveitis and macular edema among those on fingolimod 0.5 mg (Gilenya®) therapy for multiple sclerosis (MS).

**Methods:**

Retrospective review of patients on fingolimod who developed uveitis and/or macular edema.

**Results:**

No patients had an occurrence or history of uveitis. Four of the 188 (2.13%) patients developed macular edema without ocular inflammation. One of the 188 (0.53%) patients developed Acute Macular Neuroretinopathy.

**Conclusion:**

Patients taking fingolimod have a lower incidence of uveitis than expected in a population of MS patients.

## Background

Multiple sclerosis (MS) is an inflammatory demyelinating disease which targets the central nervous system in a delayed-type hypersensitivity reaction. Fingolimod (Gilenya®, Novartis) reduces both the number of relapses and progression of disease in the relapsing remitting variant of MS by preventing the migration of lymphocytes [1, 2]. Fingolimod-associated macular edema (FAME) is a known complication requiring ophthalmic examination [3–5]. Uveitis can cause vision loss and may be 20 times more likely in MS when compared to the general population [6–11]. The recommendation of pretreatment ophthalmic exams and monitoring while on fingolimod presents a unique opportunity to evaluate MS patients who may have undiagnosed uveitis and to follow for its development.

Herein we report an incidence of uveitis and macular edema among patients on the FDA-approved dose of fingolimod 0.5 mg for MS.

## Main text

### Methods

The electronic health record at The Retina Institute (St. Louis) was reviewed for patients referred for screening exams due to fingolimod use. Each case was examined for a past history, recurrence, or new occurrence of uveitis. Snellen visual acuity was reviewed as well as time of exposure to fingolimod. Optical coherence tomography (OCT) and biomicroscopy results were available for all patients. The incidence of optic neuritis, FAME, and other visually significant ocular comorbidities were also recorded.

## Results

A total of 188 patients met the study criteria over a period of 104 months. None had or developed uveitis [6–11]. One of the 188 (0.53%) developed acute macular neuroretinopathy (AMN). Twenty six of 188 (13.83%) had a history of optic neuritis and 7 (3.7%) had a new occurrence during the course of the study. The mean follow up on fingolimod was 60.9 months with a range of 1–104 months. Three patients discontinued treatment after 57, 43, and 13 months due to nonophthalmic reasons. Visual acuity (VA) was 20/40 or better in 93% of eyes, 5% had visual acuity 20/50 to 20/200, and 2% had worse VA than 20/200. The visual acuity of two eyes was worse than 20/400 as a result of optic neuritis. Additional causes of vision loss in the patients with vision worse than 20/100 were optic neuritis, ischemic optic neuropathy, ruptured globe, keratoconus, macular hole, and amblyopia.

### Fingolimod-associated macular edema (FAME)

**Fig. 1:**
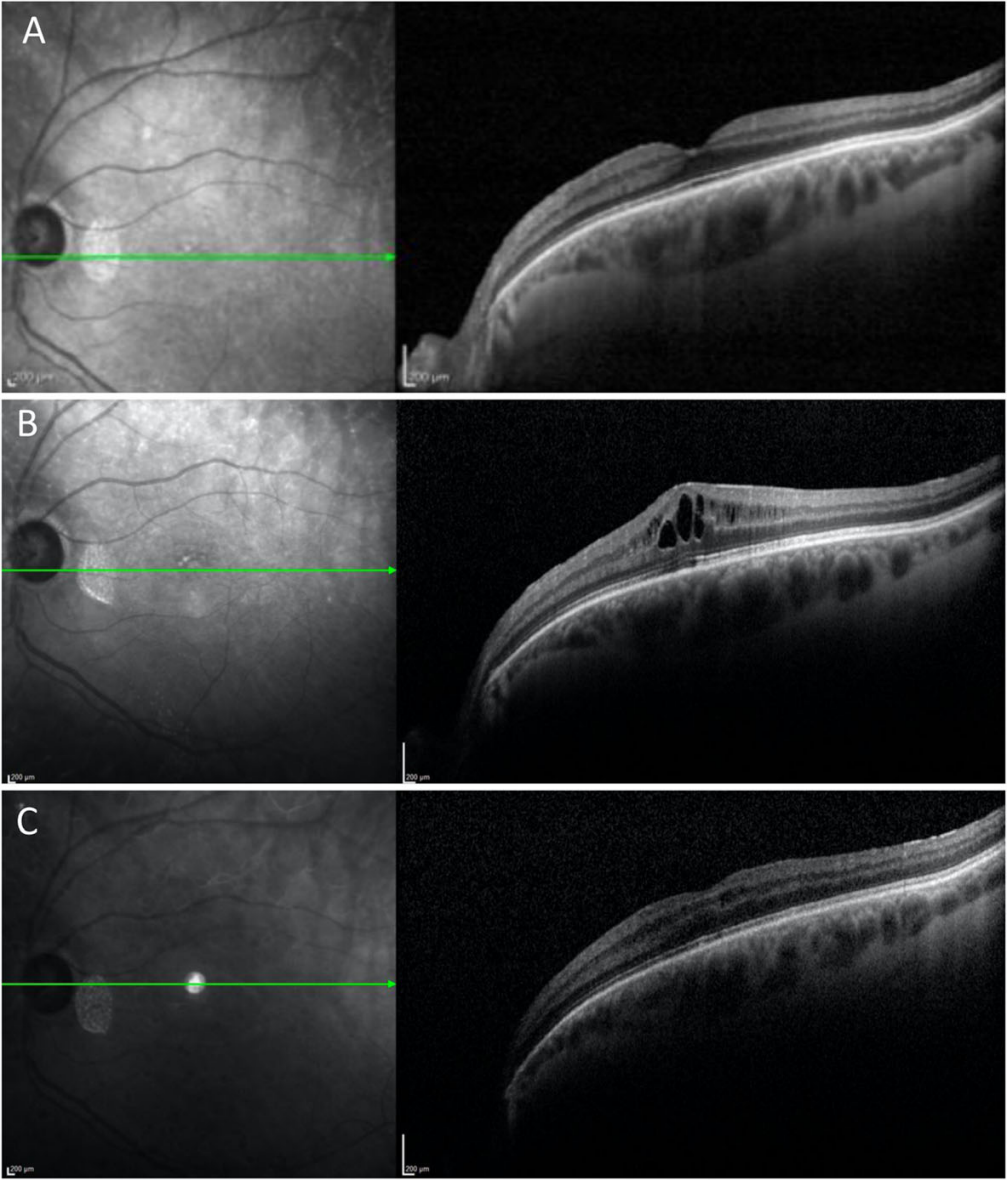
A 58 yo female with a 9 month history of fingolimod developed macular edema in her left eye. She responded to bromfenac topical drops used twice daily although she continued fingolimod. Normal macular contour is demonstrated on OCT at the initial screening visit (**a**) OCT demonstrates FAME at the 9 month follow up visit (**b**) OCT after 6 weeks shows reduction in the edema (**c**)

Three of the 188 patients (1.6%) developed FAME (Fig. 1). Fingolimod was continued and each was successfully treated with either topical steroids, NSAIDs, or a combination of the two. None of these patients had any other identifiable cause of macular edema. Another 65-year-old female with a 57 month exposure to fingolimod developed macular edema 2 weeks after cataract extraction. She was successfully treated with topical and subtenons steroids while continuing the fingolimod.

### Uveitis

No patient had a history of or developed uveitis during the follow up period.

### Other findings

**Fig. 2:**
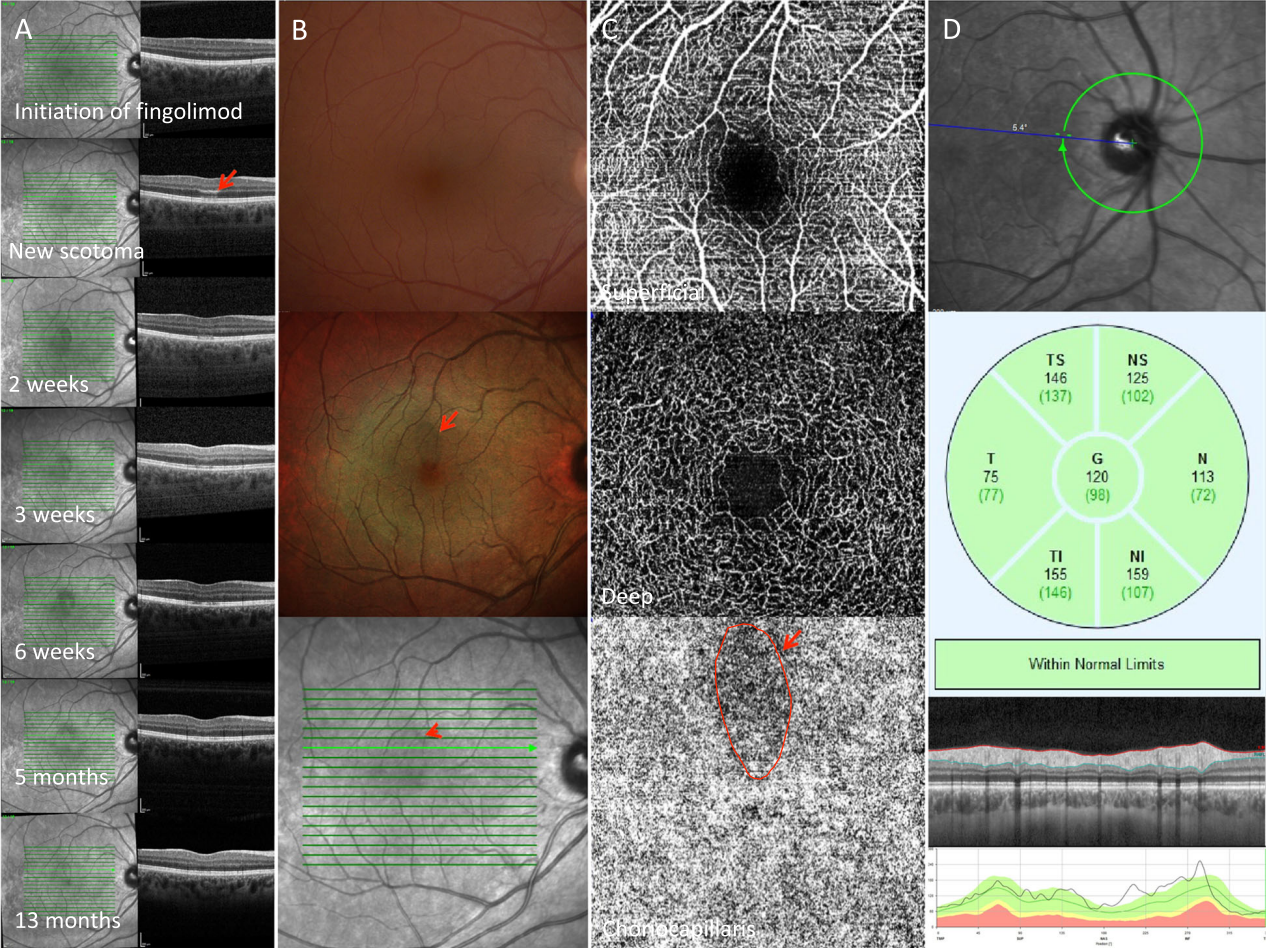
A 27 yo female on fingolimod developed Acute Macular Neuroretinopathy (AMN) 5 months after initiating treatment. Optical coherence tomography (OCT) reveals natural history of AMN, seen as hyperreflectivity of the outer plexiform layer with secondary involvement of the ellipsoid layer and external limiting membrane (**a**) Subtle reddish parafoveal lesion is shown on color fundus image yet better visualized as a petalloid lesion with multicolor imaging (**b**) Choriocapillaris OCT angiography shows a filling defect (**c**) Retinal nerve fiber layer OCT is within normal limits (**d**)

One 27-year-old female presented with unilateral photopsia and a paracentral scotoma 5 months after beginning fingolimod. Her migraines were treated with sumatriptan and she took lisdexamfetamine for weight control. Otherwise, she denied recent viral prodrome, vaccinations, pregnancy, oral contraceptive pills (OCPs), and international travel. VA was 20/20 and dilated fundus examination revealed a subtle reddish lesion just superior to the fovea of the right eye consistent with acute macular neuroretinopathy (Fig. 2). The patient’s visual defect faded and the flashes of light resolved by 5 months after presentation. She remained stable throughout her 13 months of follow up.

## Discussion

The incidence of uveitis in this cohort (*n* = 188) using fingolimod for MS was zero. The incidence of uveitis in MS patients is variable yet none in our cohort had a typical uveitis occurrence despite a mean observation period greater than 5 years [6–9, 12–16]. Lim et al. previously pooled data on the incidence of uveitis from the MS fingolimod studies [12]. The mean observation period was 627 days and included a range of fingolimod doses compared with placebo and interferon beta-1a. The authors reported a first time uveitis occurrence in 0.09% of patients on fingolimod (dose range 0.5 mg to 5 mg), 0.2% in the placebo group, and 0.09% in the interferon group. Of the 139 with a uveitis history, 5 (3.6%) had recurrence while on fingolimod. One in 8 (12.5%) patients on placebo had recurrence while 4 of 15 (26.67%) taking interferon had recurrent uveitis. Though the MS fingolimod studies were not designed to evaluate the effect of fingolimod on the prevention of uveitis or a reduction in uveitis recurrence, the data suggest those on placebo had double the rate of uveitis occurrence compared with those on fingolimod. Those taking intramuscular interferon had a similar rate of first time uveitis. The rate of recurrent uveitis in those taking a placebo was over 3 times those on fingolimod, while recurrence in those taking interferon was just over 7 times those on fingolimod. In the last 8 years since we began screening patients on fingolimod we noted an unusually low incidence of concomitant uveitis despite the diagnosis of MS. We cannot comment on the rate of recurrence since none of our cohort had a prior history of uveitis. However, this retrospective review confirms a low initial occurrence of uveitis in patients taking fingolimod for MS. Varying doses of fingolimod and interferon beta-1a were included in the data compiled by Lim et al. [12]. Our current study is unique in that it includes only the FDA-approved 0.5 mg dose of fingolimod. In addition, the cohort is a relatively homogenous group without a history of uveitis who were observed for a significantly longer period (mean > 5 years).

Fingolimod limits lymphocytic migration by targeting sphingosine-1-phosphate (S1P) receptors found in lymphocytic tissue, endothelium, and leukocytes [1, 2, 17]. It is therefore not unreasonable to hypothesize that there is a limitation of lymphocytic migration through the blood-retinal barrier as well when patients are exposed to fingolimod. There is precedence for fingolimod’s ophthalmic anti-inflammatory effect in experimental autoimmune uveitis. Murine studies have confirmed active disease suppression, maintenance of disease remission, and increased vascular barrier integrity when subjects are exposed to fingolimod [18, 19]. We hypothesize fingolimod’s anti-inflammatory effect could partly explain the lack of uveitis in our cohort that would typically be associated with MS.

One patient developed AMN. There is no consensus on a unifying mechanism of AMN though it is most strongly associated with flu-like illnesses, influenza vaccination, and OCP’s [20–25]. The patient in our cohort had other risk factors for AMN including lisdexamfetamine and migraine headaches. Lisdexamfetamine acts by facilitating the release of norepinephrine and dopamine, known vasoconstrictors. Therefore, her AMN was likely due to relative ischemia from vasoconstriction rather than a manifestation of inflammation within the retinal vasculature. The latter seems unlikely since she nor any other patient in our cohort developed typical clinical findings consistent with uveitis. There is no known direct link between AMN and MS alone, although it has been associated with acute demyelinating optic neuritis [26, 27]. In a prospective study of 114 patients with acute optic neuritis, Deschamps et al. found 6 developed AMN [27]. Therefore it remains that the development of AMN in our patient is likely coincidental since she had no history of optic neuritis.

The average time to the development of uveitis after an MS diagnosis ranges from 3.6 to 9 years [6–9]. We did not include time since the onset of MS diagnosis since this information was not available on every patient. Yet the mean follow up on fingolimod was 60.9 months and was likely adequate considering the average onset of uveitis after MS. Further, the length of time of MS was at least the same or greater than the fingolimod exposure (mean = 60.9 months) since MS was the indication for fingolimod use in all patients.

FAME occurred in 3 of the 188 (1.6%) patients in the present study. This rate is much higher than the previously documented 0.2% incidence [3, 4, 28]. The mechanism of FAME is not fully understood although it develops within 3 months from initiation of fingolimod therapy in 68% of cases [3, 4]. It has been shown to decrease vascular permeability yet it also increases tight junction permeability resulting in edema formation [1, 29]. The incidence of FAME in those with a history of uveitis was 19% compared with 1% in the FREEDOMS and TRANSFORMS trials in patients taking the 1.25 mg dose of fingolimod [29]. This data suggests a link between uveitis and FAME although there may be a variable effect on vascular permeability that is dependent on the individual’s immune system status [2, 4]. It is possible that those with a history of uveitis may have weakened tight junctions, resulting in a predilection for FAME with S1P receptor activation rather than vascular barrier stabilization [29]. The decreased dose (0.5 mg) of fingolimod now FDA-approved for the treatment of MS may not have such a high association of uveitis and FAME. Our patients with FAME had no history of uveitis or indications of intraocular inflammation, though subclinical uveitis cannot be ruled out in the absence of fluorescein angiography. There was an increased incidence of FAME in this cohort and all cases responded to anti-inflammatory therapy despite continuation of fingolimod.

The strengths of this study include a uniquely long follow up period with a mean of over 5 years, the generalizability of the findings due to exposure to the only FDA-approved dose of fingolimod for adults, and the consistency of examination by ophthalmologists with experience in the evaluation and treatment of anterior, intermediate, and posterior uveitis. No patients were excluded from taking fingolimod. However, as a retrospective review it is possible the referring pattern of the treating neurologists may have introduced some selection bias. This did not occur to our knowledge since the determination of ocular contraindication to fingolimod treatment was the responsibility of the examining ophthalmologist.

## Conclusions

Fingolimod is a well tolerated drug. Despite the high incidence of FAME in our cohort it was easily managed with topical therapy. We report a zero incidence of first time uveitis typically associated with MS in patients taking fingolimod and call for further evaluation of its potential use in the management of uveitis.

## Data Availability

Data was collected using the electronic health record of The Retina Institute in St. Louis. The datasets generated contain health protected information and are therefore not available to the public.

## Abbreviations

MS: Multiple sclerosis
FAME: Fingolimod-associated macular edema
OCT: Optical coherence tomography
AMN: Acute macular neuroretinopathy
VA: Visual acuity
OCP’s: Oral contraceptive pills
S1P: Sphingosine-1-phosphate
